# Revisiting roles of mast cells and neural cells in keloid: exploring their connection to disease activity

**DOI:** 10.3389/fimmu.2024.1339336

**Published:** 2024-03-08

**Authors:** Eunhye Yeo, Joonho Shim, Se Jin Oh, YoungHwan Choi, Hyungrye Noh, Heeyeon Kim, Ji-Hye Park, Kyeong-Tae Lee, Seok-Hyung Kim, Dongyoun Lee, Jong Hee Lee

**Affiliations:** ^1^ Department of Dermatology, Samsung Medical Center, Sungkyunkwan University School of Medicine, Seoul, Republic of Korea; ^2^ Department of Medical Device Management and Research, Samsung Advanced Institute for Health Sciences and Technology, Sungkyunkwan University, Seoul, Republic of Korea; ^3^ Department of Plastic Surgery, Samsung Medical Center, Sungkyunkwan University School of Medicine, Seoul, Republic of Korea; ^4^ Department of Pathology, Samsung Medical Center, Sungkyunkwan University School of Medicine, Seoul, Republic of Korea

**Keywords:** keloid, single-cell sequencing, mast cell, neural cell, microenvironment, cell-cell interaction

## Abstract

**Background:**

Mast cells (MCs) and neural cells (NCs) are important in a keloid microenvironment. They might contribute to fibrosis and pain sensation within the keloid. However, their involvement in pathological excessive scarring has not been adequately explored.

**Objectives:**

To elucidate roles of MCs and NCs in keloid pathogenesis and their correlation with disease activity.

**Methods:**

Keloid samples from chest and back regions were analyzed. Single-cell RNA sequencing (scRNA-seq) was conducted for six active keloids (AK) samples, four inactive keloids (IK) samples, and three mature scar (MS) samples from patients with keloids.

**Results:**

The scRNA-seq analysis demonstrated notable enrichment of MCs, lymphocytes, and macrophages in AKs, which exhibited continuous growth at the excision site when compared to IK and MS samples (*P* = 0.042). Expression levels of marker genes associated with activated and degranulated MCs, including *FCER1G, BTK*, and *GATA2*, were specifically elevated in keloid lesions. Notably, MCs within AK lesions exhibited elevated expression of genes such as *NTRK1, S1PR1*, and *S1PR2* associated with neuropeptide receptors. Neural progenitor cell and non-myelinating Schwann cell (nmSC) genes were highly expressed in keloids, whereas myelinating Schwann cell (mSC) genes were specific to MS samples.

**Conclusions:**

scRNA-seq analyses of AK, IK, and MS samples unveiled substantial microenvironmental heterogeneity. Such heterogeneity might be linked to disease activity. These findings suggest the potential contribution of MCs and NCs to keloid pathogenesis. Histopathological and molecular features observed in AK and IK samples provide valuable insights into the mechanisms underlying pain and pruritus in keloid lesions.

## Introduction

Keloids are fibrotic skin diseases that occur at areas with a cutaneous injury. They typically extend beyond boundaries of the original wound (1–8). They are characterized by excessive collagen deposition and abnormal cellular proliferation (5–9). Keloids can be pruritic near the site of injuries. They can also result in pain or restrict movement due to excessive scarring. Currently, available treatments for keloids encompass surgical excision, cryotherapy, steroid injections, and radiation therapy (10–15). Nevertheless, treatment of keloids remains challenging as they often exhibit a tendency to recur even after undergoing interventions. This highlights the existence of an unmet demand for more effective approaches that directly address underlying mechanisms involved in keloid development.

Environmental factors, including the incidence rates varying after surgery or burn injury, emphasize the influence of external triggers on keloid formation. Additionally, the differential susceptibility of individuals from various ethnic backgrounds, with dark-skinned populations exhibiting higher rates, indicates the role of environmental factors in conjunction with genetic predisposition (16–18). In recent years, there has been a significant advance in understanding molecular mechanisms underlying keloid pathogenesis (2, 19–23). While these advances have shed light on the proliferation of mesenchymal fibroblasts (FBs) driven by fibrogenic growth factors such as TGF-β signaling and their association with the pathological accumulation of extracellular matrix components, the broader landscape of keloid development remains complex (3, 9, 24, 25). It is critical to recognize the multifactorial nature of keloid pathogenesis, which includes genetic predisposition, environmental influences, and molecular mechanisms. The development of single-cell RNA-sequencing (scRNA-seq) gives us an opportunity to characterize transcriptional heterogeneity among keloid FBs. Previous studies using scRNA-seq have suggested potential roles of many other cell types including vascular endothelial cells and immune cells in keloid pathogenesis (2, 22). However, the interaction between mast cells (MCs) and neural cells (NCs) in keloids at the single-cell level has not been reported yet. To date, numerous studies have demonstrated that degranulation of activated MCs, which release chemical mediators, can induce pain by sensitizing and stimulating nociceptors and their central synaptic targets (26–28). Furthermore, substantial evidence suggests that MCs should not be regarded as mere bystanders in the process of fibrosis and wound healing (29). Recent studies have shed light on the capability of MCs to activate TGF-β1 through intragranular tryptase and chymase, thereby directly or indirectly contributing to tissue fibrosis (3, 30, 31). Consequently, MCs might also play a role in aberrant collagen production that underlies the development of keloids.

The current study aimed to investigate the role of MCs and NCs in keloid formation and to assess the therapeutic potential of targeting these cells as a strategy for treating keloids. We conducted scRNA-seq for human active keloids (AK), inactive keloids (IK), and mature scar (MS) samples, with a focus on identifying differences in expression levels of genes related to MCs and NCs. The results of this study will help us better understand molecular features of keloids based on their activity and roles of MCs and NCs in abnormal fibrosis and the resulting pain and itching.

## Materials and methods

### Patient samples

Samples from keloid patients were collected after obtaining informed consent and Institutional Review Board (IRB) approval (IRB number: SMC 2020-03-032). Patients confirmed to have clinical evidence of keloids were analyzed in this study.

Keloid samples were classified as AK and IK based on a comprehensive evaluation that included patient feedback such as itching, pain intensity, and an assessment of persistent growth for at least 6 months before and after tissue collection. AK was identified in patients who showed persistent growth at the biopsy or excision site when observed for more than 6 months, increased in size compared to the original lesion, and reported moderate or greater pain and pruritus intensity (6 or greater on a scale of 0-10). IK, on the other hand, includes patients who report no change in size at the biopsy or excision site and mild to no pain (3 or less on a 0-10 scale) when observed for at least 6 months. This classification system was implemented to capture the dynamic nature of keloid development and to distinguish between active and inactive phases. By incorporating these clinical criteria, we aimed to provide a more detailed understanding of the RNA expression patterns associated with different stages of keloid development. We collected six AK samples, four IK samples, and three MS samples from patients with keloids. Keloid samples were obtained on multiple regions via 4 mm punch biopsy or partial excision to minimize entire lesion damage. In this study, we analyzed keloid samples from chest and back regions only, excluding other areas. These samples were used to investigate the cellular microenvironment of keloid.

### Droplet-based single-cell RNA sequencing

Single-cell suspensions of keloid and scar samples were subjected to scRNA-seq using Chromium Single Cell Controller and Single Cell 3′ Library & Gel Bead Kit (10X Genomics, Pleasanton, CA, USA) in accordance with the manufacturer’s protocol.

In detail, single-cell suspensions were generated immediately after resection as previously described (31, 32). Briefly, the keloid tissue was washed in phosphate-buffered saline (PBS). Excised keloids were immersed in physiological saline and immediately transferred to the lab. After the sample was minced into small pieces with a scalpel in a Petri dish, prepared dissociation solution (2 mg/ml Liberase TL in PBS, Sigma Aldrich) was used to digest at 37°C for 60 min. The debris was filtered out with a 70-μm cell strainer (#352340, Corning). The remaining tissue was transferred into a dissociation solution containing 0.25% Trypsin for the second round of dissociation. Subsequently, the live cells were resuspended in PBS-0.04% bovine serum albumin. Cells were pelleted by centrifuging at 500 x g for 5 min. Live cells were counted and confirmed using the LUNA-FL dual fluorescence cell counter (Logos Biosystems). To generate single-cell gel bead emulsions, suspensions of skin cells were immediately injected into the Chromium Controller (10x Genomics) targeting 9,000 cells, respectively. Following the manufacturer’s protocols, the Chromium Single Cell 3’ Reagent V3.1 Chemistry Kit (10x Genomics) was used to construct single-cell libraries. Then, prepared libraries were sequenced on the NovaSeq 6000 Sequencing System (Illumina, USA) targeting 50,000 raw reads per cell.

CellRanger pipeline (10x Genomics) was used to map the sequencing reads to a human reference genome (GRCh38). The raw gene expression matrix generated from the CellRanger pipeline was processed for downstream analyses using the Seurat package version 4.2.0 in R version 4.1.1 software (R Foundation for Statistical Computing, Vienna, Austria). Downstream analysis was performed after cell quality filtering. Cells were included if they expressed greater than 500 unique molecular identifier (UMI) counts, fewer than 6,500 genes, greater than 200 genes, and fewer than 15% of mitochondrial gene expression in UMI counts. Doublet identification was performed using the tool DoubletFinder (v2.0.3) by creating artificial doublets and measuring the proportion of artificial k nearest neighbors (pANN) for each cell using PC distance. The cells were then ranked according to the expected number of doublets.

The 10x sequencing data were mapped to human reference genome (GRCh38; official Cell Ranger reference) for generating the raw gene expression matrix. The Seurat package version 4.2 was used for preprocessing and normalizing gene expression data. Genes that were expressed in fewer than 3 cells were excluded in the expression matrix. After quality control filtering, cells from multiple donors were merged using the standard integration protocol described in Seurat v4. Following the Seurat tutorial, we selected the 3000 most variably expressed genes to identify major cell types in each sample. We used the single-cell data integration method “Harmony” to correct for batch effects in scRNA-seq data sets used in this study before performing downstream analysis.

### Clustering and scRNA-seq analysis

Cells were clustered using dimensionality reduction and Uniform Manifold Approximation and Projection (UMAP) with Seurat R package. The DEGs for each cluster were calculated to ascribe an initial annotation using the Seurat function “FindAllMarkers” (Wilcoxon’s rank sum test) with default parameters. To build second level clusters, cells belonging to subpopulations were reanalyzed separately; all steps were performed using NormalizeData, FindVariableFeature, ScaleData, RunPCA, FindNeighbours, FindClusters, and RunUMAP, the methods implemented in the Seurat package for each of the major cell types. Then, second-level cluster-based doublet exclusion was performed. After doublet removal, we repeated the above mentioned steps to identify subclusters.

### Gene ontology and pathway enrichment analyses

For the gene ontology (GO) analysis, we ranked the differentially expressed genes (DEGs) for the SC-0 and SC-2 clusters. Candidate DEGs were further filtered at P-value <0.001 and average log (fold change) > 2. We used the Metascape web tool (www.metascape.org) to conduct GO analysis. The annotation dB R package org.Hs.eg.db was used to map gene identifier. Gene Set Enrichment Analysis (GSEA) was performed using R package clusterProfiler and gene sets from the Molecular Signature Database (MSigDB, gsea-msigdb.org).

To identify shared marker genes for keloids, differential expression was performed using the “FindMarkers” function implemented in Seurat with default parameters. The marker gene set was filtered to include genes with an adjusted *p*-value <0.05 and an average log fold change higher than 1.6. We further excluded genes with the ribosome, with mitochondria, and with detectable expression in >40% of normal control clusters from these shared keloid marker genes.

### Cell-cell communications analysis

The analysis was conducted using the interaction tool NicheNet to explore cell-cell communication. The definition of expressed genes as described in Puram et al. was used to determine background expressed genes in subpopulations. Briefly, NicheNet infers intercellular communication based on the ligand activities and expression of previously defined downstream targets regulated by these prioritized ligands. The function “Predict_ligand_activities” implemented in NicheNet was applied to infer ligand-target potential scores. We also applied Cellchat to infer possible cell-cell ligand-receptor interactions present within keloid cell type. Using the same clusters included for NicheNet analysis, we confirmed which ligands influence transcription during keloid development. A significant interaction was determined when the mean receptor and ligand expression of the subpopulations were significantly higher than those of subpopulations of cells determined from random permutations.

### Mast cell histochemistry

Tissues were fixed with 4% paraformaldehyde and paraffin sections were prepared. Sections were stained with hematoxylin and eosin (H&E). Toluidine blue was used to stain all mature MCs by binding to serglycin proteoglycans in secretory granules. MCs were assessed by quantifying their densities within images of toluidine blue-stained slides in ten random locations of a tissue section (magnification 20×). MC density was expressed as cells/mm^2^.

### Immunohistochemistry

All specimens were fixed in formalin and embedded in paraffin blocks; the blocks were then cut into 4-µm sections. All tissue sections were immunohistochemical (IHC) stained for the MC marker tryptase and the nerve fiber marker protein gene product 9.5 (PGP9.5). Tissue sections were incubated simultaneously with two primary antibodies: mouse anti-human MC tryptase monoclonal antibody (working dilution 1:100, Santa Cruz Biotechnology) and mouse anti-human PGP9.5 monoclonal antibody (working dilution 1:100, Bio-Rad). Sections were incubated with anti-mouse multimer labeled with horseradish peroxidase (blue staining) for Tryptase and with anti-mouse multimer labeled with alkaline phosphatase substrate (pink staining) for PGP9.5.

### Statistical analysis

All statistical analyses were performed using R4.1.1 software (R Foundation for Statistical Computing, Vienna, Austria). The Mann-Whitney U test was employed for comparing differences in means using the R program. Chi-square tests were used to compare differences in categorical variables among the three groups. Cell counts from AK, IK, and MS samples obtained via scRNAseq were compared with one another with one-way Analysis Of Variance (ANOVA) with *post hoc* Tukey’s honest significant difference tests. Statistical significance was accepted at p<0.05 for all comparisons.

## Results

### Cellular composition of keloids and matured scars

**Figure 1:**
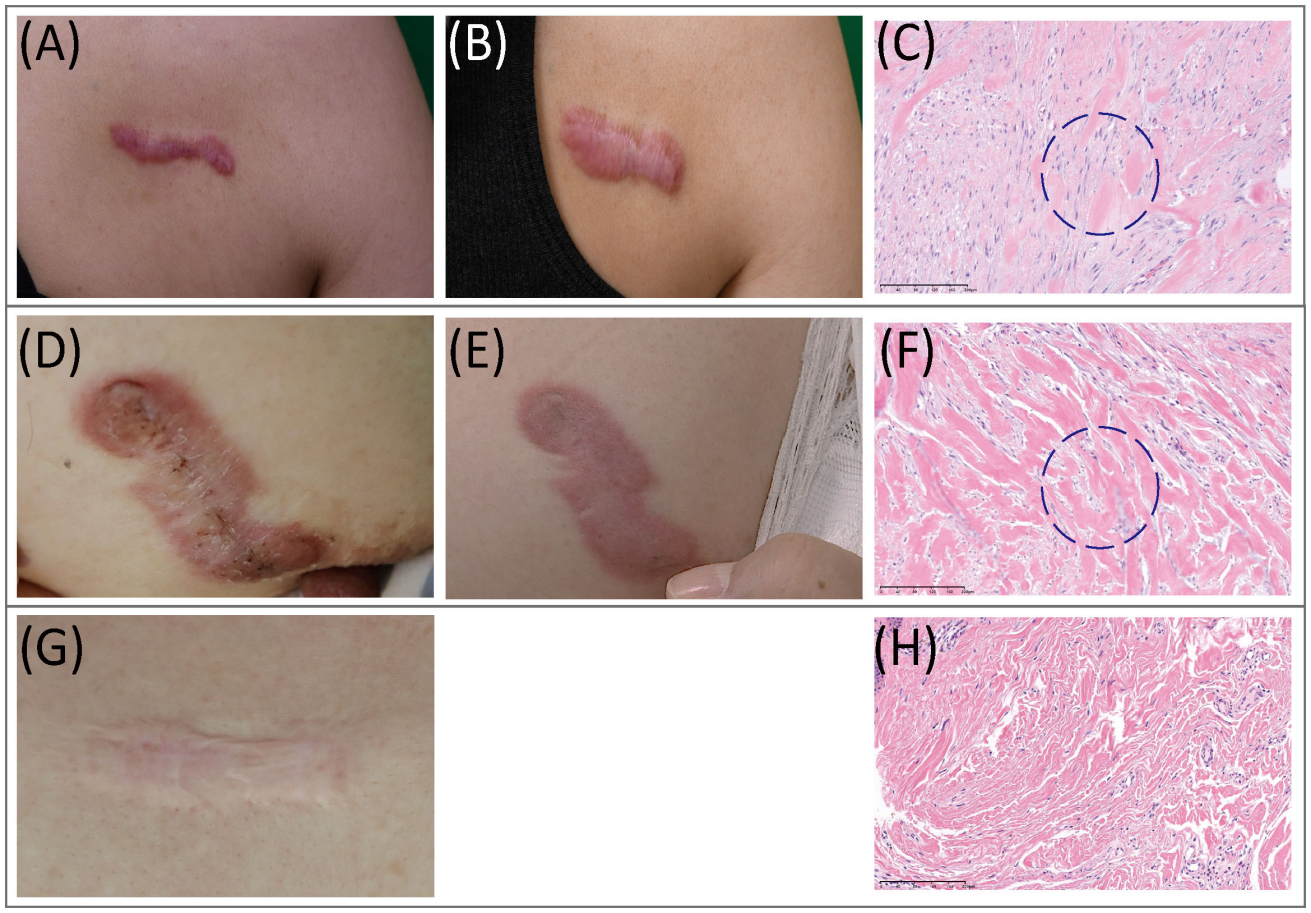
Clinical and histopathological features of keloids. **(A–C)** Clinical pictures and histopathological staining of AK. **(A)** Preoperative view; **(B)** Three months after operation. **(C)** In AK stained with H&E (20x), a heavy infiltrate of FBs and lymphocytes was found. **(D–F)** Clinical pictures and histopathological staining of IK. **(D)** Preoperative view; **(E)** Three months after the operation. **(F)** In IK stained with H&E(20x), compact collagen bundles and abundant FBs were found. **(G, H)** MS. **(G)** Clinical pictures of MS. **(H)** MS samples stained with H&E(20x). AK, active keloid; IK, inactive keloid; MS, matured scar; H&E, hematoxylin and eosin.

Histopathological features of AK, IK, and MS tissues were reviewed and evaluated by dermatologists and a pathologist. Remarkably, H&E tissue sections of AK revealed notable increases in cellular components, which were distributed amidst scattered collagens bundles (**Figure 1**). In contrast, histological analysis of IK samples showed a distinct presentation characterized by densely packed and thick collagen bundles accompanied by sparse cellularity.

To gain a more comprehensive understanding and confirm histopathological findings within the AK compared with IK, we performed a scRNA-seq of fresh keloids derived from three AK patients and two IK patients. Each keloid was divided into center and peripheral areas for the analysis. MS obtained from three keloid patients was also collected during surgery (n=3) (**Supplementary Tables S1**). We collected two MS samples each from two patients who had already been sampled for IKs.

**Figure 2:**
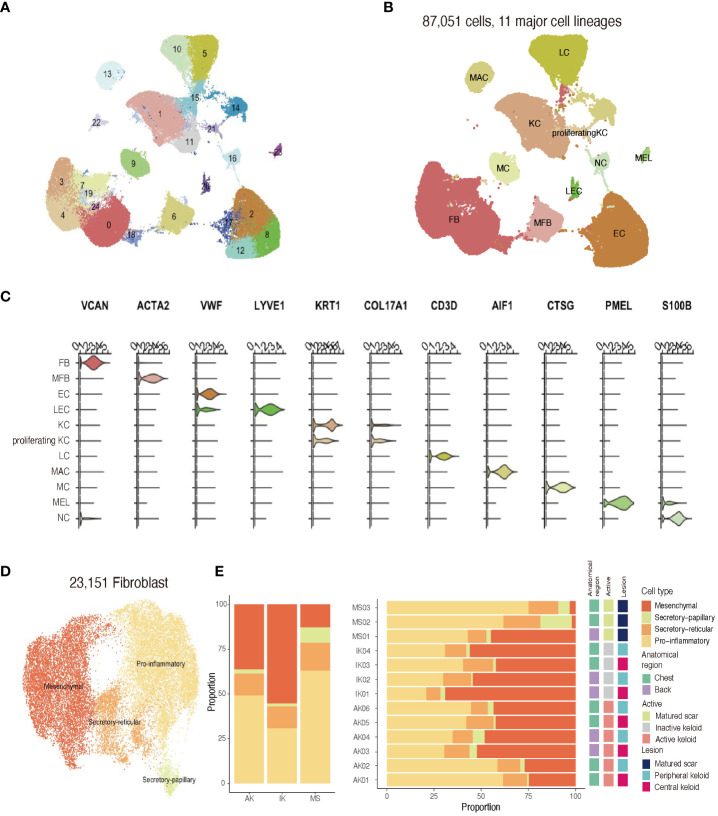
Single-cell RNA sequencing reveals cellular landscape of keloids and scars. **(A)** Uniform manifold approximation and projection (UMAP) plot depicting single-cell transcriptomes of keloids and MSs (n = 13). **(B)** A UMAP plot demonstrating 11 major cell lineages. **(C)** Cluster annotation. The violin plot shows canonical marker expressions representative of each cluster. KC, keratinocyte; FB, fibroblast; MFB, myofibroblast; EC, endothelial cell; LEC, lymphatic endothelial cell. **(D)** UMAP projection of FB subclusters. Keloid and scar FBs could be divided into four subpopulations: secretory-papillary, secretory-reticular, mesenchymal, and pro-inflammatory **(E)** Relative proportions of each subcluster of FBs in AK, IK, MS, and each sample. AK, active keloid; IK, inactive keloid; MS, matured scar.

After quality control and removal of doublets, we profiled a total of 87,051 single cells (71,617 cells from keloids and 11,161 cells from MS). All profiled cells were then clustered and visualized with Unsupervised Uniform Manifold Approximation and Projection (UMAP) (**Figure 2A**; **Supplementary Figure S1**). We then classified cells into 11 major cell types and annotated cell clusters using classic markers and particular transcriptional signatures: FB (*COL3A1*), vascular endothelial cells (EC; *PECAM* and *VWF*), lymphatic endothelial cells (LEC; *LYVE1*), myofibroblasts (*ACTA2* and *TAGLN*), lymphocytes (LC; *CD3D*), mast cells (MC; *CTSG* ^+^), macrophage (MAC; *AIF*), keratinocytes (KC; *KRT1*), proliferating keratinocytes (proliferating KC; *COL17A1* and *MKI67*), melanocytes (MEL; *PMEL*), and neural cells (NC; *S100B*) (**Figures 2B, C**; **Supplementary File S1**) (33, 34).

Recent studies have described FB heterogeneity within keloids and suggested this molecular heterogeneity is important for understanding underlying mechanisms of keloid pathophysiology (2, 21, 22). To address changes occurring during the fibrotic process in keloids, we conducted an unsupervised clustering analysis (n=23,151) of an FB subpopulation. Results of the scRNA-seq analysis revealed that FBs were divided into four subpopulations using specific markers from previous studies: secretory-papillary, secretory-reticular, mesenchymal, and pro-inflammatory FB (**Supplementary File S2**) (2, 22, 35). Next, we compared differences between keloids and matured scars obtained from keloid patients (**Figures 2D, E**). Consistent with previous findings, the proportion of mesenchymal subpopulation was increased in keloids when compared to MS. Upregulated genes identified in mesenchymal FBs were primarily associated with ECM organization and skeletal system development, suggesting their potential roles in keloid pathogenesis.

### Distinct profiles of mast cell populations between active keloids, inactive keloids, and mature scars

**Figure 3:**
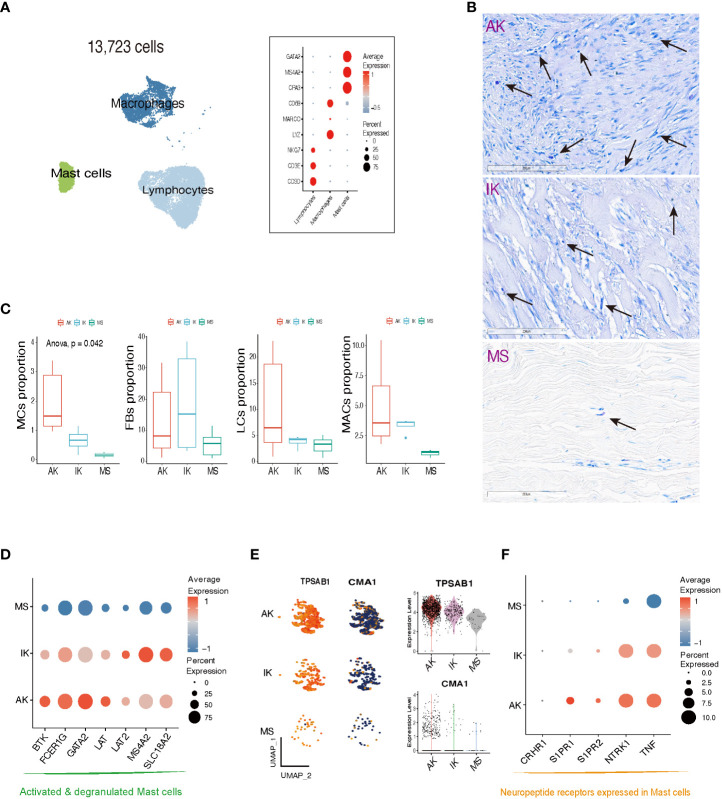
Characteristics of mast cells in AK, IK, and MS. **(A)** UMAP plot and Dot plot depicting the expression of selected marker genes for Lymphocytes (LCs), Macrophages (MACs), and Mast cells (MCs). **(B)** Toluidine blue stain showing MCs in AK, IK, and MS under high power (20x). **(C)** Box plot comparing cell compositions of each cell subset in each colored annotated group. Cochran Armitage trend test. **(D)** Dot plot showing expression of selected marker genes for MCs degranulation activation. **(E)** Feature plots, Violin plots showing expression of *TPSAB1* and *CMA1* marker genes in AK, IK, and MS. **(F)** Dot plot showing expression of selected marker genes for neuropeptide receptor.

The scRNA-seq analysis showed that immune profiles differed significantly between keloids and MSs, especially in the MC population. In line with previous studies, MCs specifically expressed *GATA2, MS4A2*, and *CPA3* (**Figure 3A**; **Supplementary File S3**) (22, 35). Interestingly, the proportion of MCs was significantly enriched in keloid samples compared to MS samples. Building upon these findings, our subsequent work focused on investigating the MC population within keloids. Toluidine blue immunohistochemical stain, a known marker for MCs, further supported the enrichment pattern of MCs in keloids. This enriched pattern was particularly dominant in active lesions of keloids (**Figure 3B**). These results were also depicted in the box plot, which illustrated MC proportions as determined by scRNA-seq analyses. Our comprehensive analyses revealed a notable abundance of both LC and MAC within AK samples. Collective enrichment of LC, MAC, and MC suggested inflammatory response and immune system activation that could be observed in AK tissues. On the other hand, FB was significantly enriched in keloids, showing a higher abundance in IK samples (*p*-value = 0.042, Cochran Armitage trend test; **Figure 3C**).

We examined the expression pattern of marker genes associated with activated and degranulated MCs, specifically *FCER1G, BTK*, and *GATA2.* We found that these marker genes were also significantly elevated in the MCs population of the keloid group compared to the MC population of the MS group, suggesting a potential association between keloid pathogenesis and stressed state of MCs (**Figure 3D**) (36–38). In addition to an increase in the number of MCs, there was an upregulation of tryptase expression and a downregulation of chymase expression within keloid tissues (**Figure 3E**). The upregulation of serine protease tryptase is particularly intriguing as it has been implicated in various functions, including induction of substance P release, activation of neurokinin 1 receptor, and amplification of inflammation, leading to thermal and mechanical hyperalgesia (39). Conversely, downregulation of chymase in skin might contribute to prolonged survival of inflammatory cytokines and neuropeptides, potentially promoting inflammation and itching in keloids.

Our further analyses revealed that MCs derived from AK and IK showed an upregulation of sphingosine 1-phosphate receptors, specifically *S1PR1* and *S1PR2*, along with an upregulation of *NTRK1*, which encodes *TrkA*. These receptors are involved in regulation of pain-related nerve growth factor signaling (39). Comparatively, MCs derived from MSs did not exhibit the same level of upregulation, whereas the highest level of expression was observed in MCs from AK (**Figure 3F**). Collectively, our findings suggest that MCs are implicated in both pain and pruritus through expression of neuropeptide receptors (40–42).

### Involvement of neural cells in keloid formation and tissue repair

**Figure 4:**
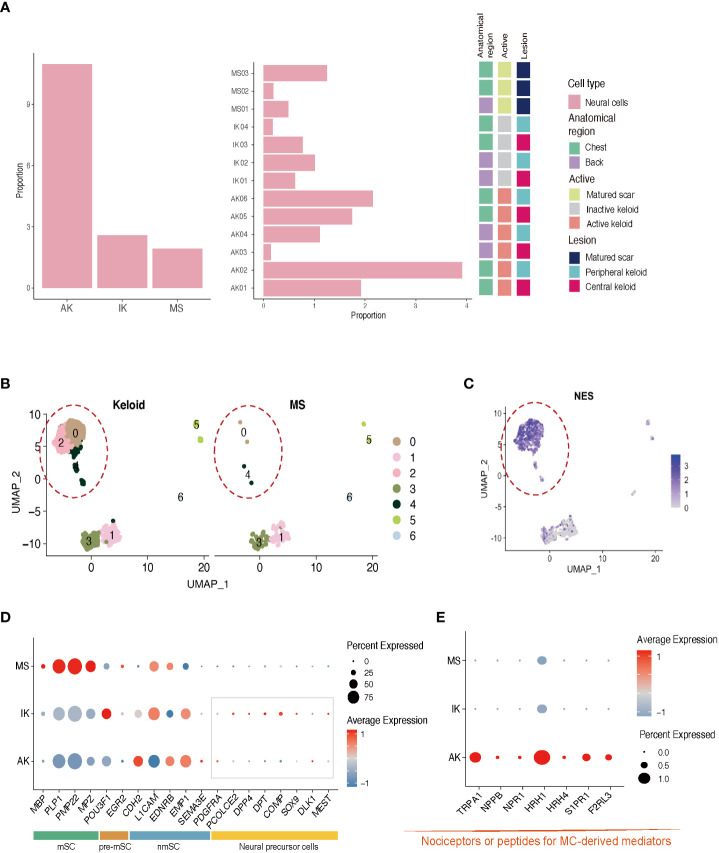
Characteristics of neural cells in AK, IK, and MS. **(A)** Relative proportions of neural cells in AK, IK, and MS samples. **(B)** UMAP plot depicting single-cell transcriptomes of keloids and MS. **(C)** Feature plots showing expression of *NES*. **(D)** Dot plots depicting the expression of mSC, nmSC, pre-mSC, and NMC in AK, IK, and MS. mSC, myelinating Schwann cells; nmSC, non-myelinating Schwann cells; pre-mSC, pre-myelinating Schwann cells; NMC, neural mesenchymal precursor cells; **(E)** Dot plot depicting the expression of selected marker genes (*TRPA1, NPPB, NPR1, HRH1*, and *HRH4, S1PR1, F2RL3*) for pain.

Next, we performed subgroup analysis focusing on the population of NCs expressing *S100B*. Consistent with previous studies, keloids showed a higher proportion of NCs than MSs (**Figure 4A**; **Supplementary File S4**) (43). Further clustering analysis of the NC population revealed the presence of four distinct subclusters. Interestingly, NC-0 and NC-2 were specifically observed in keloid samples (**Figure 4B**). Differentially Expressed Genes (44) analysis confirmed that neural progenitor cell markers such as *NES* were highly expressed in these clusters (**Figure 4C**) (43). Gene Ontology (GO) enrichment analyses revealed that clusters NC-0 and NC-2 exhibited upregulation of DEGs that were enriched in specific GO terms, including NABA CORE MATRISOME, extracellular matrix organization, and nervous system development (**Supplementary Figure S2**). These results could be supported by previous findings showing that NCs participate in signal transduction and act on changes in ECM structure to promote keloid formation (43, 45).

Expression of nmSC (non-myelinating Schwann cell) genes such as *L1CAM* and *EMP1* was observed in all lesions, whereas expression of mSC (myelinating Schwann cell) genes including *MBP* and *MPZ* was specific to MS. Interestingly, the expression of genes related to neuronal precursor cell genes, which could directly contribute to tissue regeneration and skin repair, was upregulated in the IK group (**Figure 4D**) (46, 47). These results suggest that NCs might have undergone damage during the formation of keloids and that neural progenitor cells are in the process of proliferating to repair the associated nerve damage concurrently. A more detailed analysis of NCs revealed that AK specifically expressed pain sensation-related genes such as *TRPA1* (nociception-related sodium channels), *NPPB* (itch-related neuropeptide), *NPR1* (Nppb receptor), *HRH1* (histamine receptor), and *HRH4* (**Figure 4E**) (27, 48–51). These data indicate that AK has higher expression levels of presumed unmyelinated, peptidergic nociceptors known to have the potential to induce pruritic responses in humans than IK.

To eliminate the possibility of bias, we analyzed only keloid samples from the chest and back, excluding other sites, and also observed the expression of markers between chest and back keloids, which revealed no site-specific differences. Gender-specific differences were not observed either in the analysis (**Supplementary Figure S3**).

### Analysis of ligand-receptor interaction reveals a potential role of mast cells in the formation of keloids

We investigated interactions of MCs and NCs with various cell types through the R package “cellchat” (52). Comparing keloids and MS, our data showed that cell-cell interactions between FBs and other cell types were more enriched in keloids than in MS (**Supplementary Figure S2**), consistent with previous studies (45). The number of interactions among MCs, NCs, and FBs was measured. Although the difference was not statistically significant, there was a notable increase in the number of interactions between MCs and NCs in keloids compared to MS (**Supplementary Figure S2**). These results suggest that MCs and NCs might play a role in the maintenance of keloid activation.

**Figure 5:**
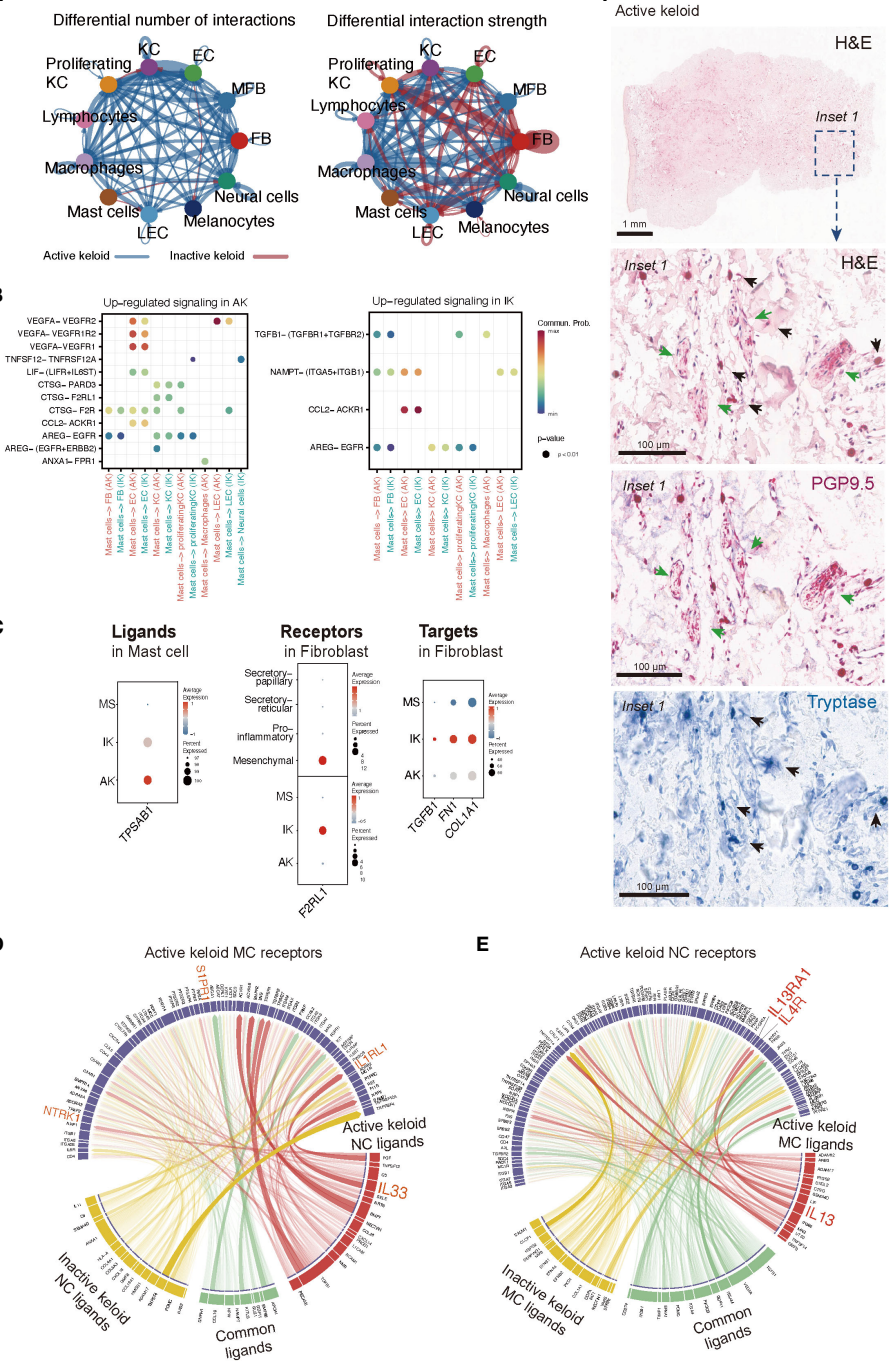
Intercellular Communication in AK and IK. **(A)** Circus plots depict differential or strength of interactions between two datasets in the cell communication network. Red (or blue) colored edges represent increased (or decreased) signaling in the IK compared to the AK. All significant signaling pathways were ranked based on their differences of overall information flow within inferred networks between IK and AK. **(B)** Comparison of significant ligand-receptor pairs between AK and IK contribute to signaling from MCs to another cell type. Dot color reflects communication probabilities and dot size represents computed p-values. Empty space means communication probability is zero. *p*-values are computed from one-sided permutation test. **(C)** A dot plot showing expression of ligand *TPSAB1* in AK, IK, and MS of MC, expression of receptor *F2RL1* in AK, IK, and MS of FB, and expression of targets *TGFB1, FN1*, and *COL1A1* in AK, IK, and MS of FB. **(D, E)** NicheNet analysis reveals ligands, receptors, and target genes that contribute to transcriptional changes in MCs and NCs following keloid disease activity. **(D)** Circus plots showing the top ligand–receptor pairs identified by NicheNet. Transparency of the connection represents the interaction strength. NC ligands are on the bottom, and MCs are on top. **(E)** Summary of MCs ligand-NCs receptor interactions. **(F)** Immunohistochemical characterization of the MC and NC in the AK (20x). Distribution of tryptase-positive MC (blue; black arrows) and PGP9.5-positive nerve fibers (pink; green arrows) and H&E staining (1x).

In our analyses of IK lesions, FBs exhibited the highest level of combined outgoing and incoming signals among all cell types examined. However, in AK lesions, enhanced interactions were observed across all cell types, highlighting the heterogeneous features associated with keloid activation (**Figure 5A**). Through analysis of cellchat, we observed significant signaling changes that could potentially drive the onset of disease pathogenesis. Specifically, in AK, ligand *VEGFA* and its receptor *VEGFR2* exhibited high activities, particularly in the signaling from MCs to ECs. On the contrary, in IK, we discovered active interactions of *TGFB1*-(*TGFBR1*+*TGFBR2*) from MCs to FBs (**Figure 5B**). We observed the expression of *TPSAB1*, which encodes Tryptase, in MCs of both AK and IK. Expression of *F2RL1*, which encodes *PAR2*, showed significant upregulation in the mesenchymal subpopulation of FBs, particularly in IK (**Figure 5C**). To explore ligand-receptor interactions in more detail, we used ligand-receptor interaction tool NicheNet (53). Using NicheNet, we explored interactions between NC and MC that were significantly enriched in AK (**Figures 5D, E**). The predicted ligands exhibiting the highly expressed in NC from AK included *IL33*, *CCL28*, and *CXCL14*. Through ligand activity analysis, we identified receptors known to be targeted by these ligands in MC, such as *NTRK1*, *IL1RL1*, and *S1PR1*. Furthermore, we mapped genes that were differentially expressed in MC of AK, revealing predicted ligands with elevated expression in MC of AK, including *TNFSF14, IL13, CTSG, CXCL2, PTGS2*, and *LIF*. In parallel, we mapped the expression patterns of receptors known to be targeted by these ligands in NC and confirmed the expression of *IL13RA1* and *IL4R* in NC. To support the potential role of these cell interactions, we used PGP 9.5 as a marker for NCs and tryptase for MCs. Through IHC staining with tryptase and PGP 9.5 antibodies, we confirmed this cell-cell interaction, demonstrating the close apposition of MCs and nerve terminals in AKs (**Figure 5F**).

## Discussion

Here, we present a single-cell transcriptomic atlas of MC and NC subpopulations in keloids according to the disease activity. By unraveling the cellular heterogeneity within keloids through scRNA-seq analyses of AK, IK, and MS samples, we identified the potential role of MCs and NCs in the context of fibrotic skin disease. Most previous studies have proposed various therapeutic targets for keloid treatment, they have not taken the impact of phenotypic differences associated with disease activity status into consideration (19–21). However, a few studies have defined keloid activity by describing changes in keloid size. They have reported increased micro vascularization in progressive keloids and decreased microvascular development in relatively stable keloids, suggesting a vascular-focused treatment for progressive keloids (54–57). These studies are consistent with our observations of distinct histopathologic and molecular features in AK, marked by increased immune cell infiltration and heterogeneity and in IK, characterized by a predominant collagen feature. These findings suggest the importance of personalized keloid treatment strategies that consider the dynamic nature of keloid progression.

In addition, our study compares keloids to MSs from keloid patients rather than normal wounds or scars, which provides a unique advantage. MSs from keloid patients serve as an internal control group, allowing us to directly compare RNA expression patterns in keloids and MSs within the same patient. This approach minimizes potential confounding variables related to individual genetic predisposition or other factors that may vary between patient groups. Therefore, analysis of MS from keloid patients strengthens the internal validity of our study and increases the relevance of our findings.

Recent studies on MCs have illuminated their diverse roles, including initiation of inflammation, facilitation of re-epithelialization, stimulation of angiogenesis, and engagement in pro-fibrotic functions (58). Indeed, among various cell populations, MCs emerged as the predominant subpopulation within AK lesions in the present study, underscoring their potential pivotal role in driving disease activity. Notably, we observed that MCs within AK lesions specifically expressed genes such as *NTRK1, S1PR1*, and *S1PR2*, which are related to receptors for cytokines, chemokines, and growth factors released by neurons. These findings agreed with previous studies suggesting that enhanced interactions between MC and neurons could contribute to neurogenic inflammation (39).

The above findings prompted to us speculate that interaction between MC and NC could potentially trigger neurogenic inflammation, possibly contributing to pain and pruritus observed in AK. In addition, we found that activation and degranulation marker genes were exclusively expressed in AK and IK. This indicates that the activation and degranulation of MC are specific to keloids rather than MS. To confirm the observed relationship between MCs and NCs, we further analyzed scRNA-seq data from NC subpopulations. We found that MC-derived mediators were upregulated in AK samples. These collective results indicate that transcriptomic profiles of NCs could provide further explanation for neurogenic inflammation-induced pain in AKs (**Figure 4E**).

**Figure 6:**
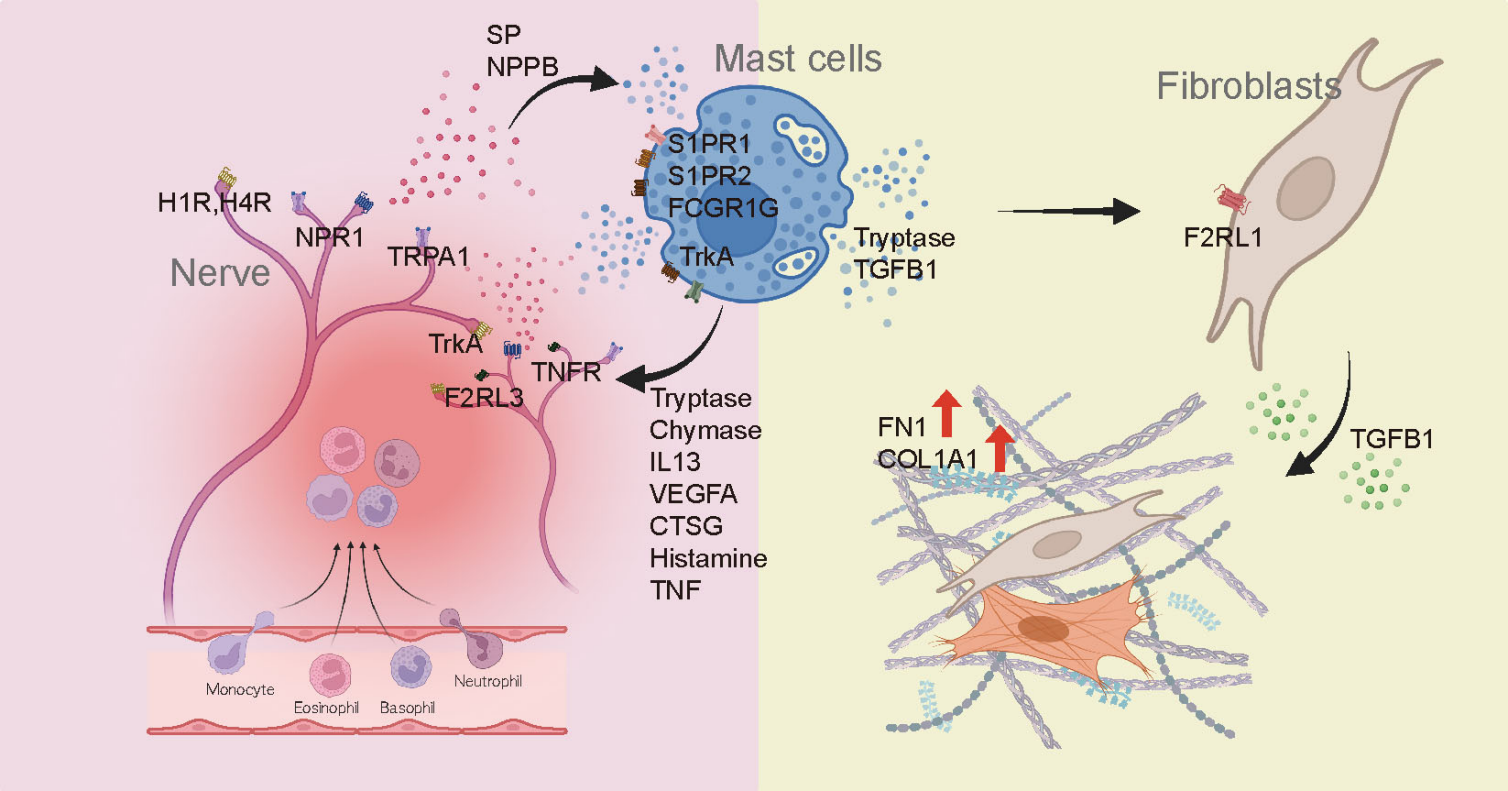
Diagram depicting the involvement of mast cells in the development of fibrosis and pain in keloids.

Recent studies have reported an association between MC and skin fibrosis in patients with secondary lymphedema and patients with idiopathic pulmonary fibrosis, highlighting the possible direct influence of MC on the progression of fibrotic conditions (59, 60). Our findings emphasize the potential involvement of MCs in fibrotic processes and provide valuable insights into the direct role of MCs in fibrosis development. Serine protease tryptase, a product of MC, has been identified as a particular point of interest in fibrosis due to its fibrogenic effects in addition to its involvement in inflammation (28, 30, 31, 61). It is predominantly manifested through activation of protease-activated receptor 2 (*PAR2*). Taken together, these findings suggest that roles of tryptase could be different according to the activation of keloids (**Figure 6**). As shown in **Figure 5C**, tryptase in MCs derived from IK might contribute to the development and intensification of fibrotic conditions of keloids, suggesting a potential association between tryptase secreted by MCs in IK and fibrotic proliferation. Thus, we propose that tryptase found in IK might act as a mitogen in the FB lineage by inducing the expression of *F2RL1* (*PAR2*) in mesenchymal FB (62–65).

Analysis of the interaction between MCs and other cell types shed light on distinct signaling pathways in AK and IK. MCs in AK were implicated in vascular proliferation and immune responses through *VEGFA* and *CTSG* signaling pathways, whereas MCs in IK appeared to regulate ECM-related subpopulations via the *TGFβ1* pathway. Although further investigation of regulators underlying MC diversity in keloids is needed, these findings highlight the need for personalized treatment approaches based on keloid phenotypes.

In conclusion, our study revealed greater heterogeneity within MCs and NCs in terms of molecular features and functions than previously expected. Although our findings suggest that AK and IK are distinct entities rather than mere similarities. Previous studies have demonstrated elevated IL-4/IL-13 expression in keloid lesions compared to normal skin, confirming the effectiveness of Th2-targeted dupilumab treatment (66–69). However, the lack of consideration for activity levels resulted in its limited efficacy across all keloid patients in clinical settings. Our study highlights the up-regulation of the IL-13 and IL-4 signaling pathways in the AK as compared to the IK. Therefore, drugs like dupilumab are anticipated to have a positive impact on alleviating pain and pruritus in patients with AK, while the use of dupilumab in IK is unlikely to be beneficial for keloid treatment. These findings contribute valuable insights into the mechanisms associated with keloid formation, paving the way for the exploration of future treatment strategies. Collectively, our results indicate that MCs and NCs are potential contributors to physiological and pathological processes in keloids. These findings could be used as a base for understanding pain and pruritus in keloids. They could also be used for identifying targets to develop effective management strategies according to the pathogenesis of keloids.

## Data availability statement

Datasets related to this article can be found at https://www.ncbi.nlm. Nih.gov/geo/using accession number of GSE220300.

## Ethics statement

The studies involving humans were approved by Samsung Medical Center, Seoul, Korea (SMC 2020-03-032). The studies were conducted in accordance with the local legislation and institutional requirements. The participants provided their written informed consent to participate in this study. Written informed consent was obtained from the individual(s) for the publication of any potentially identifiable images or data included in this article.

## Author contributions

EY: Data curation, Formal Analysis, Investigation, Methodology, Software, Validation, Visualization, Writing – original draft. JS: Data curation, Investigation, Methodology, Resources, Validation, Visualization, Writing – original draft. SO: Data curation, Investigation, Writing – review & editing. YC: Investigation, Resources, Writing – review & editing. HN: Resources, Writing – review & editing. HK: Data curation, Writing – review & editing. JP: Investigation, Resources, Writing – review & editing. KL: Resources, Writing – review & editing. SK: Resources, Writing – review & editing. DL: Investigation, Resources, Writing – review & editing. JL: Conceptualization, Data curation, Funding acquisition, Investigation, Project administration, Resources, Supervision, Validation, Writing – original draft, Writing – review & editing.

## Supplementary material

Supplementary Figure S1 Single-cell analysis of keloids revealed distinct cell populations. **(A)** Divided UMAP depicting 11 conserved cell lineages in AK, IK and MS. AK, active keloid; IK, inactive keloid; MS, matured scar; **(B)** Heatmap of differentially expressed genes in each cell lineage. FB, fibroblast; MFB, myofibroblast; EC, endothelial cell; KC, keratinocyte; LC, lymphocyte; MAC, macrophage; MC, mast cell; LEC, lymphatic endothelial cell; MEL, melanocyte; NC, neural cell; UMAP, Uniform Manifold Approximation and Projection. **(C)** Bar chart showing the proportions of corresponding cell lineages.

Supplementary Figure S2 Metascape enrichment results and changes in communication between individual pairs of cell types. **(A)** Bar plot of clusters NC-0 and NC-2 co-expression gene enrichment term. **(B)** Change in communication between individual pairs of cell types in K and MS. K, keloid; MS, matured scar; **(C)** The number of interactions or interaction strength among mast cells, neural cells, and FB. FB, fibroblast.

Supplementary Figure S3 Differences in marker expression based on keloid location and gender. **(A)** Dot plots show the expression of pain-related markers in nerve cells by keloid location and gender. **(B)** Violin plots show the expression of activated mast cell markers by keloid location and gender.
