# Supplementary figures and images for: Revisiting roles of mast cells and neural cells in keloid: exploring their connection to disease activity
Source: Front Immunol. 2024 Mar 8;15:1339336. doi: 10.3389/fimmu.2024.1339336 (PMC10957560; doi:10.3389/fimmu.2024.1339336)

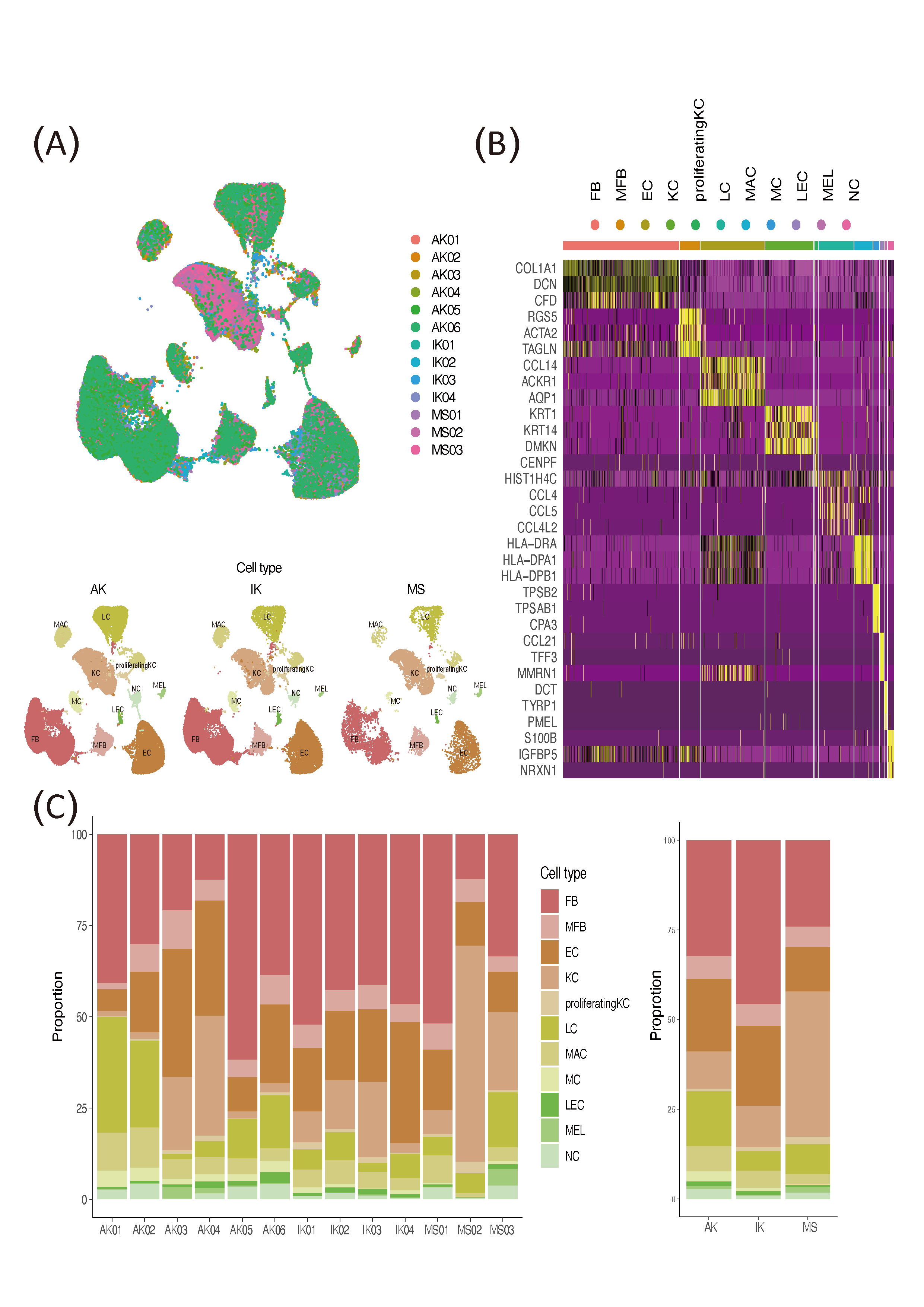

Supplement: Supplementary Figure S1 — Single-cell analysis of keloids revealed distinct cell populations. (A) Divided UMAP depicting 11 conserved cell lineages in AK, IK and MS. AK, active keloid; IK, inactive keloid; MS, matured scar; (B) Heatmap of differentially expressed genes in each cell lineage. FB, fibroblast; MFB, myofibroblast; EC, endothelial cell; KC, keratinocyte; LC, lymphocyte; MAC, macrophage; MC, mast cell; LEC, lymphatic endothelial cell; MEL, melanocyte; NC, neural cell; UMAP, Uniform Manifold Approximation and Projection. (C) Bar chart showing the proportions of corresponding cell lineages. [file Image_1.tif]

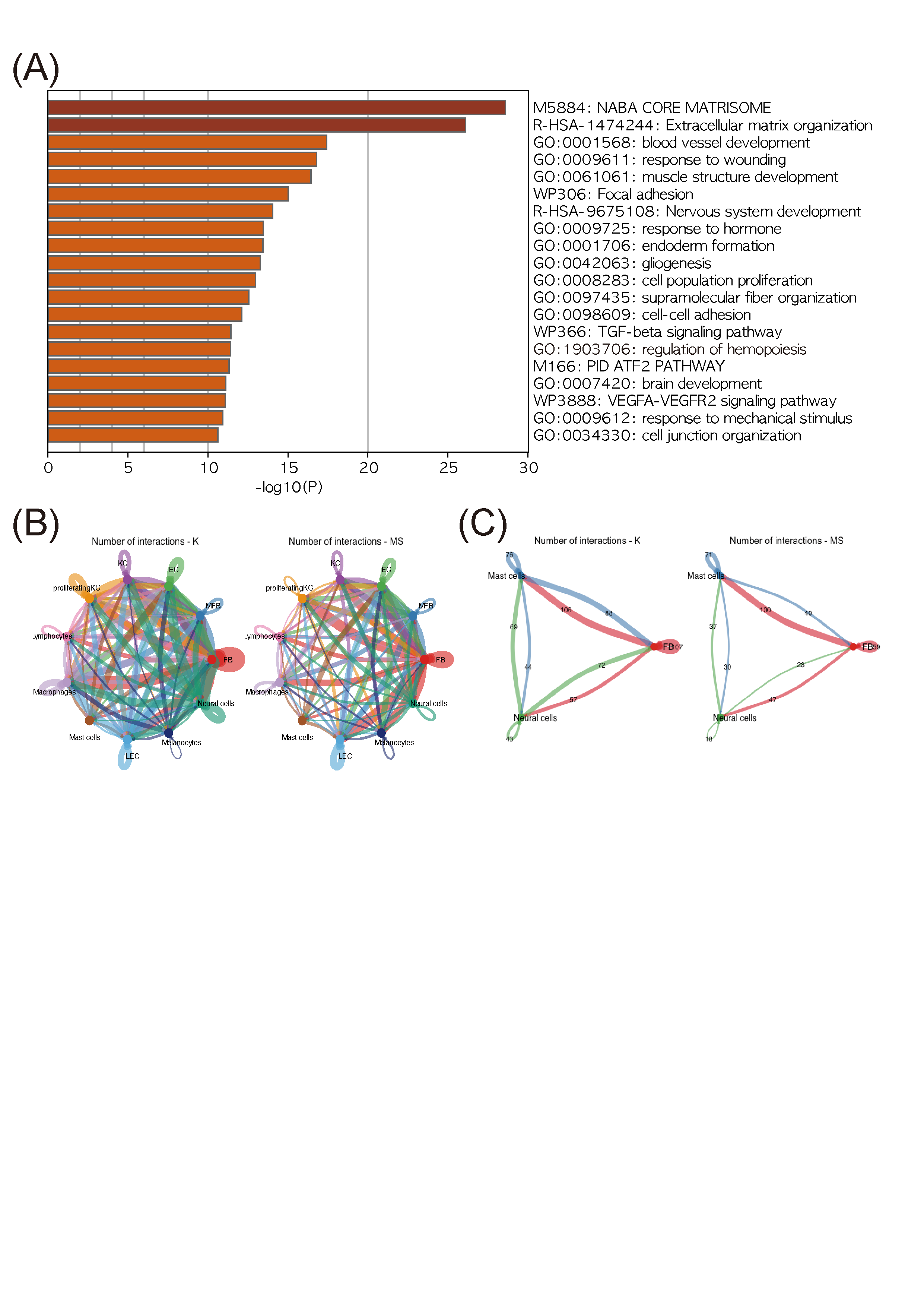

Supplement: Supplementary Figure S2 — Metascape enrichment results and changes in communication between individual pairs of cell types. (A) Bar plot of clusters NC-0 and NC-2 co-expression gene enrichment term. (B) Change in communication between individual pairs of cell types in K and MS. K, keloid; MS, matured scar; (C) The number of interactions or interaction strength among mast cells, neural cells, and FB. FB, fibroblast. [file Image_2.tif]

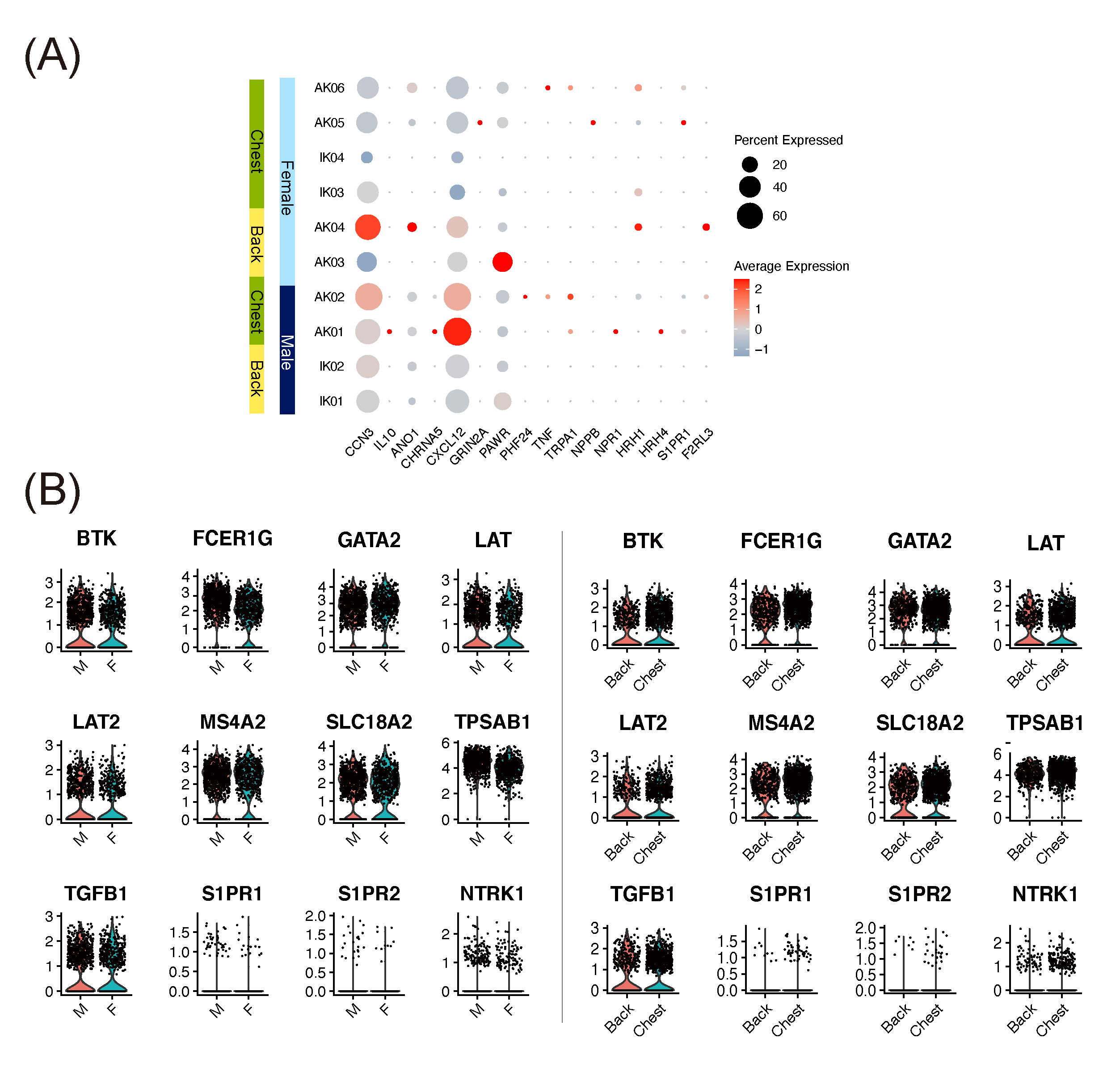

Supplement: Supplementary Figure S3 — Differences in marker expression based on keloid location and gender. (A) Dot plots show the expression of pain-related markers in nerve cells by keloid location and gender. (B) Violin plots show the expression of activated mast cell markers by keloid location and gender. [file Image_3.tif]
